# Health Worker Absenteeism in Selected Health Facilities in Enugu State: Do Internal and External Supervision Matter?

**DOI:** 10.3389/fpubh.2021.752932

**Published:** 2021-10-11

**Authors:** Divine Ndubuisi Obodoechi, Obinna Onwujekwe, Martin McKee, Blake Angell, Prince Agwu, Charles Orjiakor, Chukwudi Nwokolo, Aloysius Odii, Eleanor Hutchinson, Dina Balabanova

**Affiliations:** ^1^Department of Economics, University of Nigeria, Nsukka, Nigeria; ^2^Health Policy Research Group, College of Medicine, University of Nigeria, Nsukka, Nigeria; ^3^Department of Health Admin and Management, University of Nigeria, Nsukka, Nigeria; ^4^London School of Hygiene and Tropical Medicine, London, United Kingdom; ^5^The George Institute for Global Health, University of New South Wales (UNSW) Sydney, Sydney, NSW, Australia; ^6^Department of Social Work, University of Nigeria, Nsukka, Nigeria; ^7^School of Education and Social Work, University of Dundee, Dundee, United Kingdom; ^8^Department of Psychology, University of Nigeria, Nsukka, Nigeria; ^9^Department of Sociology and Anthropology, University of Nigeria, Nsukka, Nigeria

**Keywords:** absenteeism, supportive supervision, health workers, community health extension worker, supervisors, absent

## Abstract

**Background:** Absenteeism is widespread in Nigerian health facilities and is a major barrier to achievement of effective Universal Health Coverage. We have examined the role of internal (by managerial staff within facilities) and external (by managers at a higher level) supervision arrangements on health worker absenteeism. Specifically, we sought to determine whether these forms of supervision have any role to play in reducing health worker absenteeism in health facilities in Enugu State Nigeria.

**Methods:** We conducted interviews with 412 health workers in urban and rural areas of Enugu State, in South-Eastern Nigeria. We used binary logistic regression to estimate the role of different types of supervision on health worker absenteeism in selected health facilities in Enugu State.

**Results:** Internal supervision arrangements significantly reduce health worker absenteeism (odds ratio = 0.516, *p* = 0.03). In contrast, existing external supervision arrangements were associated with a small but significant increase in absenteeism (OR = 1.02, 0.043). Those reporting a better financial situation were more likely to report being absent (OR = 1.36, *p* < 0.01) but there was no association with age and marital status of respondents. Our findings also pointed to the potential for alternative forms of supervision, provided in a supportive rather than punitive way, for example by community groups monitoring the activities of health workers but trying to understand what support these workers may need, within or beyond the work environment.

**Conclusion:** The existing system of external supervision of absenteeism in health facilities in Nigeria is not working but alternatives that take a more holistic approach to the lived experiences of health workers might offer an alternative.

## Introduction

Absenteeism is a major problem in health systems worldwide. For example, it has been linked to the annual loss of 2 weeks of work in Organization for Economic Co-operation and Development (OECD) countries (1). However, the problem seems even greater in low- and middle-income countries, with severe consequences for already weak health systems (2–5). This is especially so in the public primary healthcare facilities on which the poor often depend. Thus, absenteeism is a major barrier to achievement of Universal Health Coverage (UHC).

Health worker absenteeism is attracting growing concern amongst service users and policy makers, concerned about the consequences for health outcomes and productivity (6, 7). This is especially so in Nigeria, where it is recognized by key stakeholders as the most important manifestation of corruption in the health system because of its widespread nature and its ability to impact service delivery and other health outcomes (8). A Nigerian study of 242 health workers found that 110 had at least one spell of absence in a year (9), while qualitative research finds it to be pervasive (5, 10, 11). Other research has pointed to lack of, or weak policies, including on supervision, the topic of this paper, as a major contributor (10).

The Covid-19 pandemic has extremely strained health workers involvement in providing health care all over the world. However, in Nigeria it didn't contributed much to absenteeism of health workers as most of them were very present at work delivering various health care to patients while protecting themselves. This is expected because by their profession, it is an obligation for them to be present at their places of work even if their health is at risk. During this study, most of them were present and work various shifts to meet up with various health care demands. Nevertheless, various PPE were provided to keep health workers safe at all times during the pandemic.

Within facilities, absenteeism has profound consequences for everyone involved. Those health workers who are present face extra work; they may have to perform tasks above their level of competence; facilities may depend on volunteers to provide services, and ultimately, patients are offered low-quality care, if they receive any at all (5, 11). As more health workers can be absent from work without facing severe consequences, those who are diligent in their work become increasingly frustrated and may, with time, engage in absenteeism (12). Health workers expressed basically that most of them are affected by negative pressures from unavoidable causes such as ill health, long distances to health facilities, family responsibilities, leadership style of their superiors, political connections among others (13). Financial pressures necessitating workers to keep a second job is also a major reason for absenteeism among health workers. The phenomenon of dual practice of health workers is a key driver to absenteeism, hence holding two or more jobs concurrently as a means to meet family demands and also make up for low salaries (14). For all these reasons, there is a pressing need to understand factors that could reduce absenteeism by health worker.

Among these factors, much attention has focused on the quality and nature of supervision, which influences the productivity and quality of care in PHCs more generally (12, 15, 16). However, what literature exists focuses on comparisons between supportive and abusive supervision (17, 18). In the current study we examine the association with absenteeism with supervision of health workers by internal health facility managers and by external supervisors, who often come unannounced. We consider these two dimensions to explore the proximity and perceptions toward the supervisor (internal vs. external) and how they contribute toward reducing absenteeism.

Supervisors support Community Health Extension Workers (CHEWs) by explaining their roles, ensuring they have the supplies needed to perform their duties effectively, and addressing any community and personal problems they encounter (19). While there is a consensus in the literature on health worker absenteeism that improved supervision is needed, evidence on its impact has been inconsistent. One study found that external supervision had mixed influences as some workers (62%) perceived it to be helpful in, amongst other things, improving supplies, identifying expired drugs, and providing on-the job training, yet other workers (24%) found external supervisors to be uninterested in the problems of the facility, making only infrequent visits (20). Hence, poor supervision may be as ineffective as none (21). Crigler et al. (19) reported how supervision had evolved from punitive and critical of those being supervised to being facilitative or supportive. However, they also differentiated facility-based supervision and that by district level supervisors. Mukasa et al. (22) in researching experiences of health workers in Uganda reported now some supervisors are perceived to be aloof and disconnected from the realities in the health center, providing little feedback (23).

While numerous studies have examined the role of supportive and abusive/punitive supervision, there is a scarcity of studies that examine whether the location of the supervisors influences the commitment of health workers to their jobs. Countries in Sub-Saharan Africa face dire shortages of health workers so it is important to understand the factors that can motivate health workers to stay at work (22). As supervision features prominently in the literature as a contributor to absence, we ask how its nature contributes to absenteeism of health workers and how this intersects with the financial situation of those health workers.

## Materials and Methods

### Study Area

This study was conducted in 10 local government areas in Enugu State, in the Southeastern part of Nigeria. The areas were purposively selected to cover urban, rural, and peri-urban areas. The population of the state is estimated at over 3 million, with 2,235,540 in rural areas and 1,032,297 in urban areas (24).

### Study Design and Population

The survey was designed to understand the nature of absenteeism in various health facilities in Enugu state and also the role supervision plays in tackling increasing rates of absenteeism in the facilities. Data were analyzed using Binary Logistic Regression Model.

The study population comprises resident doctors, nurses, midwives, and Community Health Extension Workers (CHEWs) in various health facilities across the State. Face to face interviews were conducted and at least 2 health workers from each facility were included in the study. In all 412 respondents participated in the survey from about 125 health facilities in Enugu State Nigeria.

### Data Collection

A survey instrument was designed to assess absenteeism amongst health workers and their preferences in relation to supervision. The instrument was developed following a draft instrument pretested to ascertained the views of health workers about absenteeism and potential remedies. The draft instrument was tested with 30 health workers and, after incorporation of amendments and corrections, a final version was prepared. It was converted into electronic form for use with the Open Data Kit (ODK) on an android platform.

We categorized absenteeism using two questions, one about engaging in absenteeism; and one on not engaging in absenteeism. Supervision was assessed using questions about being supervised internally by colleagues of higher rank within the facility (internal supervision) and meeting an external supervisor who comes to check health workers' activities in the facility (external supervision).

Approval to undertake the study was provided by the Enugu State Primary Health Development Agency (ESPHDA). The survey was conducted from May to June, 2020. Four researchers participated in the data collection process and were assisted by four research assistants. Heads of the (Health) Department (HODs) in all the local governments were also informed about the study and gave approval after confirming the approval of ESPHDA. HODs also provided comprehensive lists of all health centers in their local government areas from which a convenience sample of 10 PHCs was selected. Officers-in-charge (OICs) of the selected facilities were also approached with the approvals from the HODs and ESPHDA, which asked them to grant the researchers access to their staff. The survey instruments were interviewer-administered and the researchers recorded the responses on paper and in electronic media. Before leaving each site, data from both records were cross-checked and discrepancies checked with the health worker concerned. The electronic data were then uploaded to a database. The approach taken, which did involve duplication of data entry, was necessary because of COVID-19 restrictions. Researchers ensured that all safety protocols were adhered to, using facemasks and hand-sanitizers for themselves and respondents and social distancing.

### Data Analysis

The hypotheses were tested using the Binary Logistic Regression Model. Odds ratios were estimated to determine the impact of the independent variables on whether respondents reported being absent in the past year. We chose this approach because it performs very well when datasets are linearly separate from each other and it also uses the maximum likelihood robust estimation, allowing for non-normality that could be present in the data. The absenteeism variable was adopted to ascertain the variables that determine health workers' absence from work separately for the two measures. That is engaging in absenteeism and also not engaged in absenteeism.

### Variables

#### Dependent Variable: Absenteeism

We captured the effect of absenteeism in the questionnaire by asking questions about whether a health worker engaged in absenteeism (missing either a full or partial day of work over the past year) and whether they do not engage in absenteeism. Respondents who answered “yes” to being engaged in absenteeism were coded “1” and those who responded “no” were coded “0.”

#### Independent Variables

##### Met External Supervisor

This variable was included in the model to capture the role external supervision plays in regulating absenteeism in the health facilities. It captures the number of times a health worker meets an external supervisor over a set period of 1 year who monitors their work at the facility. This external supervisor could come from the local government headquarters; within the community (paramount rulers, health facility committee members, youth and women leaders, etc.); WHO (25); UNICEF; some non-government organizations, etc.

##### Performance Supervised Internally

This variable represents internal supervision of health workers by senior/higher ranking health workers in the facility. Respondents who answered “yes” to being supervised by a senior/higher ranking health worker were coded “1” and those who responded “no” were coded “0.”

##### Marital Status

This variable represents whether a health worker is married, single, divorced or separated. This was included to examine whether married health workers more frequently absent due to family commitments. Respondents who answered “single” for any of these were coded “0,” those who responded “married” were coded “1,” those who responded “divorced” were coded “3,” those who responded “separated” were coded “4.” During the data analysis process, we only used respondents who answered “married” as equal to 1 and others 0. The reason for this is that only very limited number of respondents were separated or divorced. A code was indicated in the analysis to single out only married and single in the analysis.

##### Financial Situation

This variable captures the financial situation of health workers. The hypothesis is that when a health workers' financial situation improves, they tend to be absent from work by engaging in other income generating practices, so as to earn more income. The variable was classified into 5 categories, representing “very poor” “poor” “neither good nor bad” “very good” and “good.”

### Ethical Considerations

Ethical approval was obtained from the Research Ethics Committee of the University of Nigeria Teaching Hospital (UNTH). Other approvals have been described above. The study was explained to the health workers who were given written material containing details of confidentiality and anonymity and they were asked to sign consent forms on paper and in the electronic device.

## Results

Table 1 describes characteristics of respondents. The vast majority were females and within the age group of 41–50 years (40.5%). Most of the health workers were married (79.4%). Just over a quarter (29.6%) considered their financial situation to be relatively poor, and about the same number (29.1%) relatively good.

**Table 1:** Socioeconomic and demographic characteristics of respondents.

**Variables**	**Frequency** ***N* = 412**	**Percentage**
**Gender**		
Female	400	97.09
Male	12	2.91
**Marital status**		
Single	53	12.86
Married	327	79.37
Divorced	3	0.73
Separated	2	0.49
Widow	27	6.55
**Age group**		
<30	41	9.95
30–40 years	126	30.58
41–50 years	167	40.53
More than 50 years	78	18.93
**Financial situation**		
Very poor	51	12.38
Poor	122	29.61
Neither good nor bad	107	25.97
Good	120	29.13
Very good	12	2.91
**Designation**		
Not OIC	275	66.75
OIC	137	33.25

*OIC, Officer in charge*.

Table 2 shows that 92 health workers reported never engaging in absenteeism within a year, while 320 health workers engaged in absenteeism. Absenteeism was broken down by number of days a health worker was absent from work within a 1 year and it was found that, while 92 of them never engaged in absenteeism, 225 of them were absent in 10 days or below (54.6%). Fifty-eight of them were absent for 11–20 days within a year (14.1%), 18 (4.4%), and 19 (4.6%) of them were absent between 21 and 30 days and above 30 days, respectively.

**Table 2:** Levels of absenteeism.

**Variables**	**Frequency** ***N* = 412**	**Percentage**
Engaged in absenteeism	320	77.7
Not engaged in absenteeism	92	22.3
**Absenteeism by No. of days**		
0 days	92	22.3
10 days and below	225	54.6
11–20 days	58	14.1
21–30 days	18	4.4
Above 30 days	19	4.6

Table 3 showed the correlation matrix of variables of interest. Absenteeism was positively related to financial situation with about 12.79% correlation among them. While other variables in the model had a positive correlation with absenteeism, only performance supervised internally had a negative correlation with absenteeism at about −12.1%. This is evident in the binary logistic results presented in Table 4, which also shows a negative and significant relationship with absenteeism.

**Table 3:** Correlation matrix.

	**Absenteeism**	**Financial situation**	**Performance supervised internally**	**Met external supervisor**	**Age**
Absenteeism	1				
Financial situation	0.1279	1			
Performance supervised	−0.1213	−0.0190	1		
Met external supervisor	0.0989	−0.0604	−0.0311	1	
Age	0.0645	0.0804	−0.3725	0.0299	1

**Table 4:** Descriptive Analysis of the variables used in the model.

**Variable**	**Obs**	**Mean**	**Std. dev**.	**Min**	**Max**
Age	412	42.41	8.566	21	60
Female	412	0.029	0.168	0	1
Performance supervised internally	412	0.692	0.462	0	1
Met external supervisor (number of visits per year)	405	12.11	19.56	0	169

The results in Table 4 represents the descriptive statistics of some of the variables of interest. Met external supervisor had 405 responses with a mean value of 12.1 visits per year with a standard deviation of 19.56. The maximum value of 169 represents that highest times a health worker meets an external supervisor within a year of working in the facility. Performance supervised internally represented a mean value of 0.692 meaning that 69% of respondents reported being supervised internally and a standard deviation of 0.462.

Table 5 presents the binary logistic regression results. Internal supervision (performance supervised internally) has a significant and negative relationship with absenteeism, such that those supervised internally (performance supervised internally), were 51% less likely to report engaging in absenteeism over the past year. In contrast, external supervision was positively related to absenteeism. The more health workers reported meeting external supervisors, the more likely they were absent from work. Table 4 shows a positive and significant relationship between absenteeism and meeting an external supervisor. A unit increase in meeting external supervisor led to a 2% increase in the likelihood of reporting absenteeism over the past year. There was a positive and statistically significant relationship between a health worker's perceived financial situation and absenteeism. A better financial situation is associated with more absenteeism. About 1.36% were more likely to report engaging in absenteeism over the past 1 year due to increases in their financial situation. Age and being married were both found not to be statistically associated with the absenteeism.

**Table 5:** Results of the Binary Logistic Model. Dep. Var. Absenteeism.

**Variable**	**Odds ratio**	**Std. dev**.	***Z***	***P*** **-value**
Age	1.0095	0.01639	0.59	0.558
Married	0.9523	0.14736	−0.32	0.752
Financial situation	1.3624	0.15667	2.69*	0.007
Performance supervised internally	0.5126	0.15889	−2.16**	0.031
Met external supervisor	1.0255	0.01275	2.03**	0.043
Constant	1.4217	1.15502	0.43	0.665

Source: output from STATA 15 ** and *significant at the 5% level and 1%, respectively.

## Discussion

We compared two forms of supervision, internal and external. We found that internal supervision was associated with reduced absenteeism amongst health workers in PHCs. This lends credence to other studies that obtained similar results (18, 26). External supervision was found to increase absenteeism among health workers slightly. Hence, internal supervision seems to reduce health worker absenteeism. In contrast, the association with external supervision was insignificant.

It could be that even if external supervision does not reduce absenteeism, it could play a role if it is not frequent, compromised, or previously announced. Although Onwujekwe et al. (5) finds external supervision to be important in optimizing health service delivery in PHCs, we found that it had barely any impact. There are several possible reasons. First, staff found to be absent by the external supervisor may not be punished because they are either politically connected, related, or can offer bribes. Second, external supervision was infrequent and announced so health workers would know when they will be checked and can make sure they are at work. Third, external supervisors, such as health facility committee members, community leaders, and non-government organizations had little power to enforce sanctions against health workers, notwithstanding the few exceptions recorded in the study. Coincidentally, Onwujekwe and colleagues found some of the supposed external supervisors, particularly those at the local government headquarters, were absent themselves. Though the political complexities at the local government level seem to have caused primary healthcare governance to be weak, the need to stimulate strong facility leadership could be a favorable start-point.

Our analysis shows that as a health worker's financial situation improves, they are more likely to be absent. Other research suggests that this may be because their improved financial situation allows them to open private clinics where they spend much of their time (14). However, our data did not differentiate the different sources of greater income, including higher salaries, so we cannot explore this further. Agwu et al. (11) discovered that local government health workers in Nigeria whose financial conditions are currently discouraging might abandon their responsibilities at the facilities if they can generate more income from their private businesses. To them, it is survival (27).

Despite the merits of our study, there are some limitations. We could not capture presenteeism, where those who are present are doing nothing. This was deliberate as our pilot study showed that respondents either answered in the negative or refused outright to answer. Also, we lacked questions that could address the source of the respondents' improved financial conditions. Therefore, we recommend that future studies should consider addressing these limitations.

In conclusion, since we found that external supervision provided no meaningful reduction in absenteeism, the government should explore new approaches. An uncompromised system of external supervision that is unannounced and frequent offers potential benefits. We understand that supervision is one of the most challenging ways of tackling absenteeism because of the economic and time costs for supervisors and their agencies, but there are things that can be done. First, community groups could be involved in monitoring the activities of the health workers and try to understand where such workers need help in the course of their jobs. They might become more involved in the care of the workers, feeding back ideas on how to make the work environment more attractive for workers. We identified a need to empower community-based supervisors, and other groups of external supervisors from reputable agencies and organizations to enable them to impose meaningful sanctions against healthcare staff who are absent without reason. The government could also encourage a peer support structure where a supervisor could meet regularly with groups of community health extension workers to find ways in which they can offer mutual support.

## Data Availability Statement

Data sets will be available to readers on request. Requests to access these datasets should be directed to Divine Ndubuisi Obodoechi divine.obodoechi@unn.edu.ng.

## Author Contributions

OO, DO, CN, PA, CO, and AO contributed to conception and design of the study. BA, DO, OO, and CN organized the database. DO and CN performed the statistical analysis. DO wrote the first draft of the manuscript. OO, PA, CO, AO, and CN wrote sections of the manuscript. All authors contributed to manuscript revision, read, and approved the submitted version.

## Conflict of Interest

The authors declare that the research was conducted in the absence of any commercial or financial relationships that could be construed as a potential conflict of interest.

## Publisher's Note

All claims expressed in this article are solely those of the authors and do not necessarily represent those of their affiliated organizations, or those of the publisher, the editors and the reviewers. Any product that may be evaluated in this article, or claim that may be made by its manufacturer, is not guaranteed or endorsed by the publisher.
